# Trans-Septal Myocardial Biopsy in Hypertrophic Cardiomyopathy Using the Liwen Procedure: An Introduction of a Novel Technique

**DOI:** 10.1155/2021/1905184

**Published:** 2021-02-10

**Authors:** Chao Han, Mengyao Zhou, Rui Hu, Bo Wang, Lei Zuo, Jing Li, Shengjun Ta, David H. Hsi, Jiani Liu, Lichun Wei, Liwen Liu

**Affiliations:** ^1^Department of Ultrasound, Xijing Hypertrophic Cardiomyopathy Center, Xijing Hospital, Fourth Military Medical University, Xi'an, Shannxi, China; ^2^Department of Radiation Oncology, Xijing Hypertrophic Cardiomyopathy Center, Xijing Hospital, Fourth Military Medical University, Xi'an, Shannxi, China; ^3^Heart & Vascular Institute, Stamford Hospital, Stamford, CT, USA

## Abstract

**Objective:**

The purpose of this study was to evaluate the feasibility and safety of myocardial biopsy using a new approach, the Liwen procedure.

**Background:**

Myocardial biopsy is essential when other methods could not differentiate other etiologies from hypertrophic obstructive cardiomyopathy (HOCM). Our previous work using intramyocardial radiofrequency ablation for hypertrophic obstructive cardiomyopathy (Liwen procedure) may provide another approach to obtain the myocardial samples.

**Method:**

Seventeen patients with HOCM were enrolled for biopsies through percutaneously accessed intramyocardial septum and evaluated possible complications.

**Results:**

We obtained 31 specimens from 17 patients with a success rate of sample acquisition 100.0%. The number of myocardial samples taken per patient was 1.8 ± 0.8, and the average length of all samples was 16.7 ± 5.6 mm which could be used for pathological diagnosis. The complications included pericardial effusion with and without tamponade in one patient (5.9%), and no incidence of nonsustained and sustained ventricular tachycardia, conduction abnormity, perforation, stroke, and pneumothorax. The inhospital and 30-day mortality was 0%.

**Conclusion:**

This study has shown that myocardial biopsy of the Liwen procedure is relatively safe and technically feasible with adequate tissue sampling, which may help pathological diagnosis and further research of HOCM of diverse etiologies. This trial is registered with NCT04355260.

## 1. Introduction

Myocardial biopsy should be considered when the results of other clinical assessments suggest myocardial infiltration, inflammation, or storage disease that cannot be confirmed from hypertrophic obstructive cardiomyopathy (HOCM) [1–3]. Generally, endomyocardial biopsy (EMB) sampled the subendocardial region of the right interventricular septum and the specimens.

Our previous study on the Liwen procedure, which is a nonsurgical approach for percutaneous intramyocardial septal ablation treating HOCM, may provide a new technique for myocardial biopsy [4, 5]. Myocardial biopsy of the Liwen procedure (LMB) could obtain the specimens before the radiofrequency ablation.

We developed myocardial biopsy needle of the Liwen procedure. Seventeen patients with HOCM were enrolled for the procedure. We documented biopsy results and complications.

## 2. Materials and Methods

### 2.1. Patient Population

The Institutional Ethics Committee of Xijing Hospital approved the procedure, which was performed in accordance with the ethical standards of the Declaration of Helsinki. All patients registered at clinicaltrials.gov (NCT04355260) and signed informed consent to proceed with LMB.

### 2.2. Equipment

**Figure 1:**
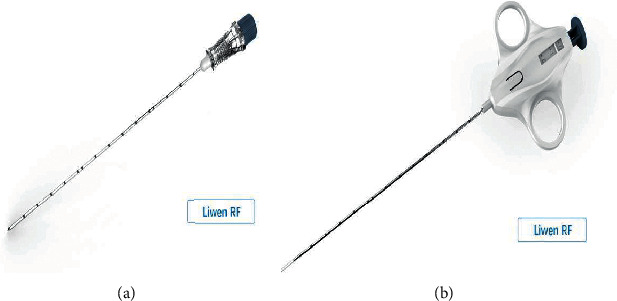
Myocardial biopsy needle of the Liwen procedure. The biopsy needles were 1.27 mm in diameter with adjustable front-end lengths of 10 mm and 20 mm, respectively. (a) Cardiac puncture sheath. (b) Cardiac biopsy needle.

Puncture sheath and cardiac biopsy needle (Figure 1) were designed and manufactured by Hangzhou Nuocheng Medical Company. The biopsy system can be used multiple times. Cardiac biopsy needle is 1.27 mm in diameter. The front-end biopsy segment is adjustable, with lengths of 10 mm and 20 mm, respectively. Transthoracic echocardiography (TTE) guidance was performed with the EPIQ 7C Ultrasound System (Philips Medical Systems, Bothell, Washington) with a 1.0- to 5.0-MHz transducer.

### 2.3. Procedure

**Figure 2:**
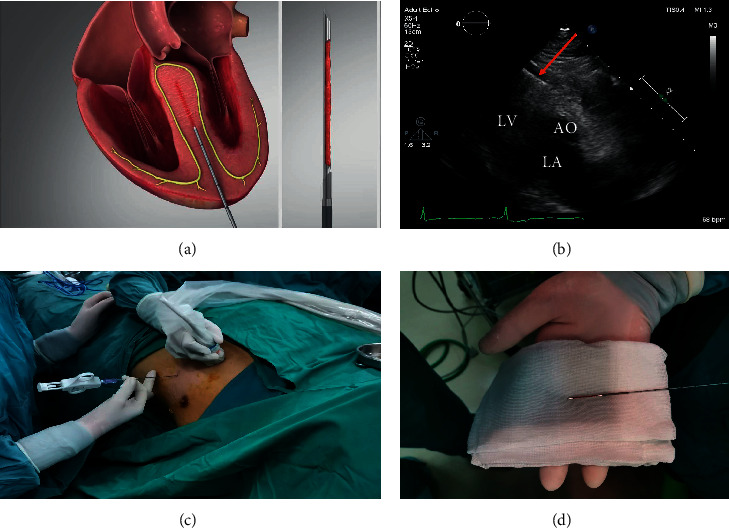
Myocardial biopsy process of the Liwen procedure. Under the guidance of echocardiography, the biopsy needle was inserted into the puncture sheath from the apex to the central septum and took biopsies. (a) LMB illustration. (b) Echocardiographic image during LMB. (c) The process of LMB. (d) The biopsy needle with specimen.

After general anesthesia, the patient was placed in the left semidecumbent position to fully expose the precordial area. Electrocardiogram tracing, blood pressure, blood oxygen levels, and central venous pressure were monitored throughout the operation. TTE-guided LMB is shown in Figure 2. Under the guidance of echocardiography, the puncture point was located at the apex of the heart, and the guide line was along the long axis of the interventricular septum. First, the puncture sheath was inserted into the hypertrophied ventricular septum. Next, about 2 cm from the predetermined biopsy position, we inserted myocardial biopsy needle into the sheath and pushed the inner core 2 cm forward until the inner switch was fired and the inner core was automatically obtained with biopsy tissue. After the cardiac biopsy needle was withdrawn to take out the myocardial tissue, ablation needle was then inserted to the same sheath to start myocardial tissue ablation. The whole process of biopsy or ablation did not enter any cardiac chamber. We documented the biopsy results, and the patients were followed up for one month.

### 2.4. Specimens

The specimens were stained with Hematoxylin-Eosin(H-E) and Congo red and analyzed by an experienced pathologist.

## 3. Results

### 3.1. Baseline Characteristics

Seventeen patients (mean age, 49.9 ± 15.2 years; 5 female patients) with HOCM were enrolled, and the baseline characteristics are shown in Table 1. The mean septal thickness was 23.7 ± 4.6 mm, mean LVOT peak gradient was 134.0 ± 54.3 mmHg, and mean ejection fraction was 58.6 ± 3.9%.

### 3.2. Liwen Myocardial Biopsy (LMB) Results

**Figure 3:**
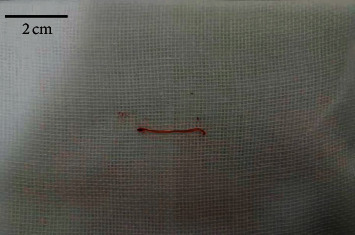
Biopsy specimens. The specimens were in the shape of red thin filament.

We obtained 31 specimens from 17 patients with successful sample acquisition in all patients (Table 2). The number of myocardial samples taken per patient was 1.8 ± 0.8, the average tissue length was 16.7 ± 5.6 mm, and the diameter was about 1.0 mm. The specimens were obtained on the first attempt and were in the shape of red thin filaments (Figure 3).

### 3.3. Complications

Pericardial effusion occurred in the eighth patient after the biopsy and was drained by percutaneous catheter for total volume of about 100 ml. No patients experienced pericardial tamponade, nonsustained or sustained ventricular tachycardia, conduction abnormity, perforation, stroke, and pneumothorax. No patients died in hospital and during the 30 days after biopsies (Table 2).

### 3.4. Pathology Diagnosis

**Figure 4:**
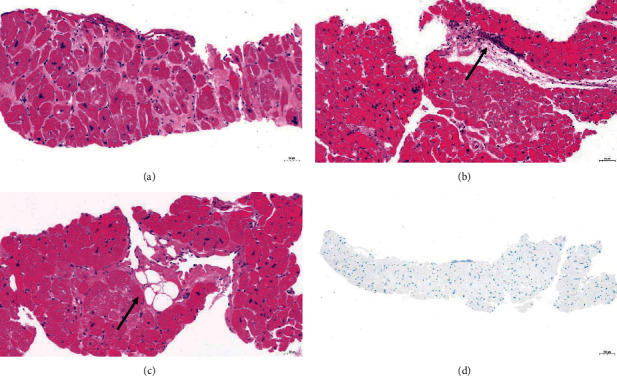
Histopathology changes in HOCM specimens. (a) Myocytes hypertrophy and hyperchromatic nuclei with bizarre shapes. (b) Inflammatory cells infiltration. (c) Adipocyte infiltration. (d) No amyloidosis (H-E staining and Congo red staining 20x).

Myocytes showed hypertrophy with an increase in the transverse diameter and hyperchromatic myocyte nuclei with bizarre shapes (Figure 4(a)). Almost all the specimens showed interstitial fibrosis. Myofiber disarray was not seen in most slides. Furthermore, inflammatory cells or adipocytes infiltration existed in some sections (Figures 4(b) and 4(c)). All the slides of Congo red staining were negative (Figure 4(d)).

## 4. Discussion

The method of Liwen myocardial biopsy (LMB) was similar to the percutaneous approach in the early development of myocardial biopsy but obtained tissue samples at the intramyocardial septum in patients with HOCM. Percutaneous needle biopsy was first studied by Sutton et al. [6]. The biopsy sites were ventricular free wall, apex [7–9], or septum through the left ventricle [10]. Due to cardiac tamponade and pulmonary complication, this procedure was abandoned in 1980s. LMB was technically feasible obtaining sufficient sample size in all patients. Sutton et al. used the modified Terry needle to make biopsies on the surface of the left ventricle and took 150 biopsy specimens from 54 patients, among which the specimens of 13 patients were not satisfactory to make diagnosis [7]. The size of samples was 3 × 1 mm. Raffensperger performed percutaneous needle biopsies on 48 patients and found that the volume of samples was insufficient to allow viral, bacteriological, microscopic, and biochemical analysis [8]. Shirey used thin-walled Silverman needle for the apical left ventricular biopsy in 198 patients and obtained adequate samples measuring 15 × 1 × 1 mm in 192 patients [9]. For our first patient, the length of the LMB was small about 2 × 1 × 1 mm because of the lack of experience by the operator. We obtained tissue sample approximately 16.7 × 1 × 1 mm in subsequent patients with 100% success rate.

We feel that Liwen myocardial biopsy is relatively safe. One patient had pericardial effusion in a pattern of slow oozing, not brisk arterial bleeding, and required percutaneous pericardial drain without further problems. We considered the main reason to be excessive movement of the biopsy needle within the myocardium trying to find the appropriate position. We need to reduce the occurrence of pericardial effusion by minimizing the needle movement. There were no patients suffered from pericardial tamponade. We did not observe any nonsustained and sustained ventricular tachycardia, conduction abnormity, perforation, stroke, and pneumothorax.

As shown in Figure 2(a), the needle was away from the conduction system distributed underneath the endocardium, so no arrhythmia occurred. Under the guidance of an experienced echocardiographer, the biopsy needle did not enter any cardiac chamber, and thus, myocardial perforation risk was negligible.

The H-E and Congo red staining of tissue samples showed histopathological characteristics consistent with hypertrophic cardiomyopathy [11]. There was no evidence of cardiac amyloidosis.

## 5. Conclusions

Our study showed that Liwen myocardial biopsy is relatively safe and technically feasible with adequate tissue sampling, which may help pathological diagnosis and further research in HOCM of diverse etiologies.

## 6. Limitations

The study population was small, and the evaluation of the feasibility and safety was preliminary. Further enrollment of appropriate patients will continue in our HCM center.

## Figures and Tables

**Table 1:** Baseline patient characteristics (*n* = 17).

	Value
*Demographics*	
Age (years)	49.9 ± 15.2
Male/female	12/5

*Echocardiography*	
Maximal septal thickness (mm)	23.7 ± 4.6
LVOT peak gradient (mmHg)	134.0 ± 54.3
Ejection fraction (%)	58.6 ± 3.9

LVOT: left ventricular outflow tract. Continuous variables are presented as mean ± SD.

**Table 2:** Results and complications of LMB(*n* = 17).

	Value
*Results*	
Number of myocardial samples taken per patient	1.8 ± 0.8
Number of total myocardial samples/total trials	31/31
Success rate of biopsy (%)	100%
Length of samples (mm)	16.7 ± 5.6

*Complications*	
Pericardial effusion with tamponade	0 (0%)
Pericardial effusion without tamponade	1 (5.9%)
Nonsustained ventricular tachycardia (≥3 ventricular complexes)	0 (0%)
Sustained ventricular tachycardia	0 (%)
Cardiac conduction abnormity	0 (0%)
Cardiac perforation	0 (0%)
Stroke	0 (0%)
Pneumothorax	0 (0%)
Inhospital and 30-day mortalities	0 (0%)
Total percentage of complications	5.9%

## Data Availability

The data used to support the findings of this study are available from the corresponding author upon request.
